# Infrared-induced variation of the magnetic properties of a magnetoplasmonic film with a 3D sub-micron periodic triangular roof-type antireflection structure

**DOI:** 10.1038/srep08025

**Published:** 2015-01-26

**Authors:** Junlong Tian, Wang Zhang, Yiqiao Huang, Qinglei Liu, Yuhua Wang, Zhijian Zhang, Di Zhang

**Affiliations:** 1 State Key Laboratory of Metal Matrix Composites, Shanghai Jiao Tong University, 800 Dongchuan Road, Shanghai, 200240, P. R. China; 2 Department of Prosthodontics, Shanghai Jiao Tong University, 800 Dongchuan Road, Shanghai, 200240, P. R. China; 3 Jushi Fiberglass Research Institute, Zhejiang Key Laboratory for Fiberglass Research, Jushi Group Co., Ltd., Zhejiang, 314500, P. R. China

## Abstract

A carbon-matrix nickel composite magnetoplasmonic film with a 3D sub-micron periodic triangular roof-type antireflection structure (SPTAS) was fabricated via a simple and promising method that combines chemosynthesis with biomimetic techniques. The *Troides helena* (Linnaeus) forewing (T_FW) was chosen as the biomimetic template. The carbon-matrix Ni wing fabricated via electroless Ni deposition for 6 h (CNMF_6h) exhibits enhanced infrared absorption. Over a wavelength range (888–2500 nm), the enhancement of the infrared absorption of CNMF_6h is up to 1.85 times compared with the T_FW. Furthermore, infrared excitation induces a photothermal effect that results in variation in the magnetic properties of the carbon-matrix Ni wing. The magnetic properties were also confirmed using atomic force microscopy (AFM) and magnetic force microscopy (MFM). The good correlation between the AFM and MFM images demonstrates that the surface of the SPTAS of CNMF_6h exhibits strong magnetic properties. The infrared induced photothermal effect that results in magnetic variation is promising for use in the design of novel magnetoplasmonic films with potential applications in infrared information recording and heat-assisted magnetic recording.

Magnetoplasmonic materials, which exhibit a combination of magnetic and plasmonic properties, have recently attracted enormous interest^1^,^2^,^3^,^4^,^5^,^6^. This interest stems from the intertwined magnetic and plasmonic characters of these materials that manifest in a wide variety of physical phenomena and enable the design of new classes of systems that exploit these novel functionalities^7^,^8^,^9^,^10^,^11^,^12^. For example, upon the excitation of the characteristic surface plasmon resonances of such materials, their plasmonic systems enhance the light-harvesting capability of the material and effectively concentrate the electromagnetic field in the magneto-optically active material to enhance the magneto-optical effects^13^,^14^. Such magnetoplasmonic materials could find applications in telecommunications, magnetic field sensing and all-optical magnetic data storage^15^,^16^.

Thus far, most studies into the magneto-optical effects of magnetoplasmonic materials have focused on the Faraday effect (rotation of the polarization of transmitted light) and the Kerr effect (rotation of the h polarization of reflected light)^8^,^14^. Additionally, the majority of the incident light sources used to induce the magneto-optical effects investigated in these experiments have wavelengths in the ultraviolet and visible ranges to match the plasmon resonances located in these wavelength ranges^8^. To our knowledge, the literature contains few reports of the infrared magneto-optical effects or discussions of magnetic variation caused by an infrared photothermal effect.

For small interparticle distances, adjacent plasmon resonances of particles hybridize and form a red-shifted bands, a relatively narrow band in the near-infrared (NIR) region and a very broadband in the mid-infrared (MIR) region^17^. However, the self-assembly of these so-called plasmon resonance nanoparticles (NPs) into thin films and bulk materials, particularly with a functional sub-micron structure, is difficult using traditional technologies such as spontaneous epitaxial nucleation^18^,^19^. To combine magnetic and plasmonic functionalities, we fabricated a carbon-matrix nickel composite magnetoplasmonic film (CNMF) with a 3D sub-micron periodic triangular roof-type antireflection structure (SPTAS) using a simple and promising method that combines chemosynthesis with biomimetic techniques and uses the *Troides helena* forewing (T_FW) as a biomimetic template. Because the black T_FW possesses an effective visible light trapping capability due to the melanin/chitin composite contained in this SPTAS^20^,^21^,^22^. The CNMF exhibits enhanced infrared (IR) absorption over a broad wavelength range. Simultaneously, the infrared excitation induces a photothermal effect that results in magnetic variation, which is promising for use in the design of novel magnetoplasmonic films with potential applications in IR information recording and heat-assisted magnetic recording.

## Results and Discussion

**Figure 1:**
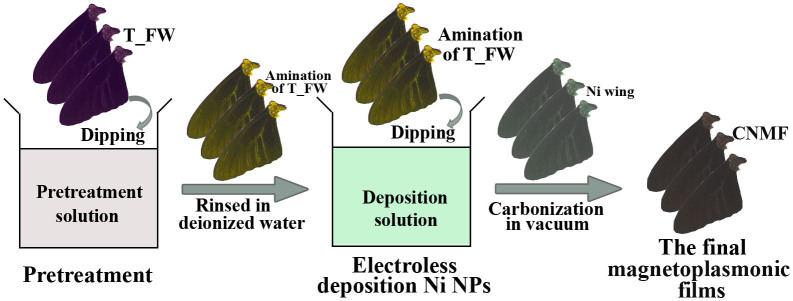
Schematic view of the fabrication of CNMF.

Figure 1 illustrates the strategy employed for fabricating the CNMF. The fabrication route described herein consists of three steps: 1) Amination of the chitin-matrix surface; 2) Standard electroless deposition of the Ni NPs onto the scales of the T_FW at 50°C. The deposition was performed over a period of 6 h to obtain the chitin-matrix Ni wing (chitin-matrix Ni wing_6h). The thickness of the Ni NP layers deposited onto the T_FW can be controlled by changing the deposition time (Figure S1)^23^; 3) Carbonization of the chitin-matrix Ni wing using a vacuum tube furnace heated to 450°C at 3°C·min^−1^ for 1 h under vacuum conditions to produce the final CNMF (CNMF_6h).

**Figure 2:**
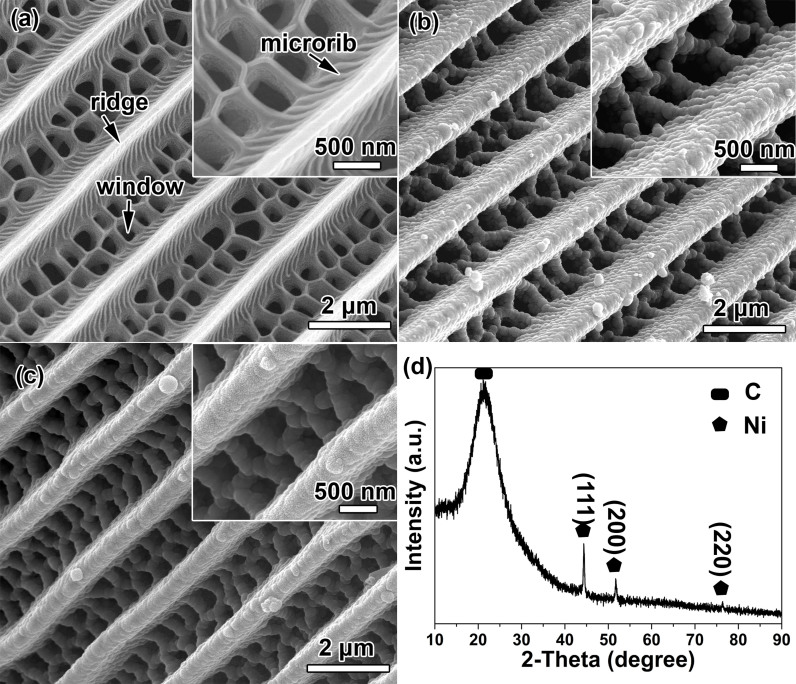
SEM images of (a) T_FW, (b) chitin-matrix Ni wing_6h and (c) CNMF_6h, respectively; (d) XRD pattern of CNMF_6h. Insets show morphologies at higher magnification.

The morphology of the T_FW, chitin-matrix Ni wing_6h and CNMF_6h were investigated using SEM (Figure 2 (a)–(c)). The morphology of the T_FW is shown in Figure 2(a). The periodic triangular roof-type ridges run the length of the scale of the T_FW and form the periodic antireflection structure that focuses light into the scale interior. Declining microribs run down the sides of the ridge that induce the internal light scattering, which assists in trapping light. Staggered windows are present between every two ridges, which imply an important linkage between this feature and the light-harvesting capacity. The ridges, microribs and windows construct a SPTAS that effectively traps light. The combination of a melanin/chitin composite with this SPTAS endows the black T_FW with an effective visible light trapping capability^20^,^21^. The SEM image of chitin-matrix Ni wing_6h clearly shows that the Ni NPs deposited onto the surface and assembled into a film that successfully inherited the SPTAS of the T_FW scale (Figure 2(b)). In our previous work^24^, we fabricated a series of Cu scales by depositing Cu NPs for different lengths of time ranging from 5 min to 25 min and observed that the Cu NPs aggregated to a greater degree with increasing deposition time. This finding confirmed that the deposition time can be utilized to control the morphologies of replicas^23^,^24^. The observed dependence of the Ni deposition thickness on the deposition time also demonstrated that the former could be controlled by the latter^25^. Accordingly, we fabricated samples subjected to deposition times of 1 h, 6 h and 10 h to demonstrate that the deposition time can be used to control the thickness of the Ni NP layer deposited onto the T_FW and thereby to control the morphologies of the Ni butterfly wings. When the deposition time was increased to 10 h (chitin-matrix Ni wing_10h), the Ni NPs coarsened and agglomerated into a thicker layer, as shown in Figure S1(c), in which the windows diminished, the ridges enlarged, and the microstructures were almost filled in by Ni NPs. In contrast, a shorter deposition time of 1 h induced the formation of a discontinuous layer of Ni NPs on the surface of the SPTAS (Figure S1(a)). Consequently, the thickness of the Ni NP layer deposited onto the T_FW and the morphologies of the Ni wings were controlled via the electroless plating time. After carbonization, the SPTAS of the T_FW scale was also perfectly transferred to the carbon-matrix structure (Figure 2(c)). As shown in Figure 2(b) and (c), compared with chitin-matrix Ni wing_6h, almost no changes arose in the morphology of CNMF_6h. The successful deposition of the Ni NPs onto the surface of the carbon-matrix SPTAS of the T_FW scale was confirmed, as shown in Figure S2, by EDS analysis. The XRD pattern of the CNMF_6h is presented in Figure 2(d). The peak located at 21.12° indicates that the carbon in CNMF_6h is amorphous and the peaks located at 44.52°, 51.70° and 76.37° were indexed as the (111), (200) and (220) planes of the cubic phase of Ni (JCPDS no. 04-0850), respectively.

**Figure 3:**
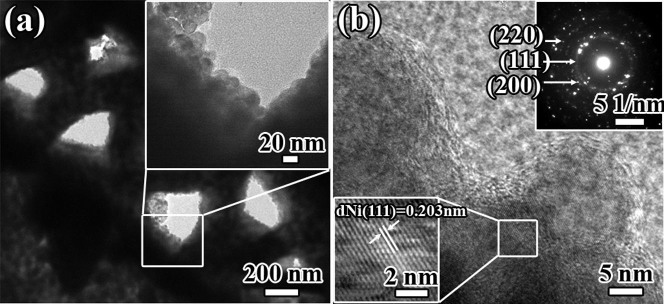
(a) TEM image of CNMF_6h. The inset of (a) shows the morphology at higher magnification. (b) The HRTEM image of CNMF_6h. The insets of (b) are the SAED image and HRTEM image, respectively.

**Figure 4:**
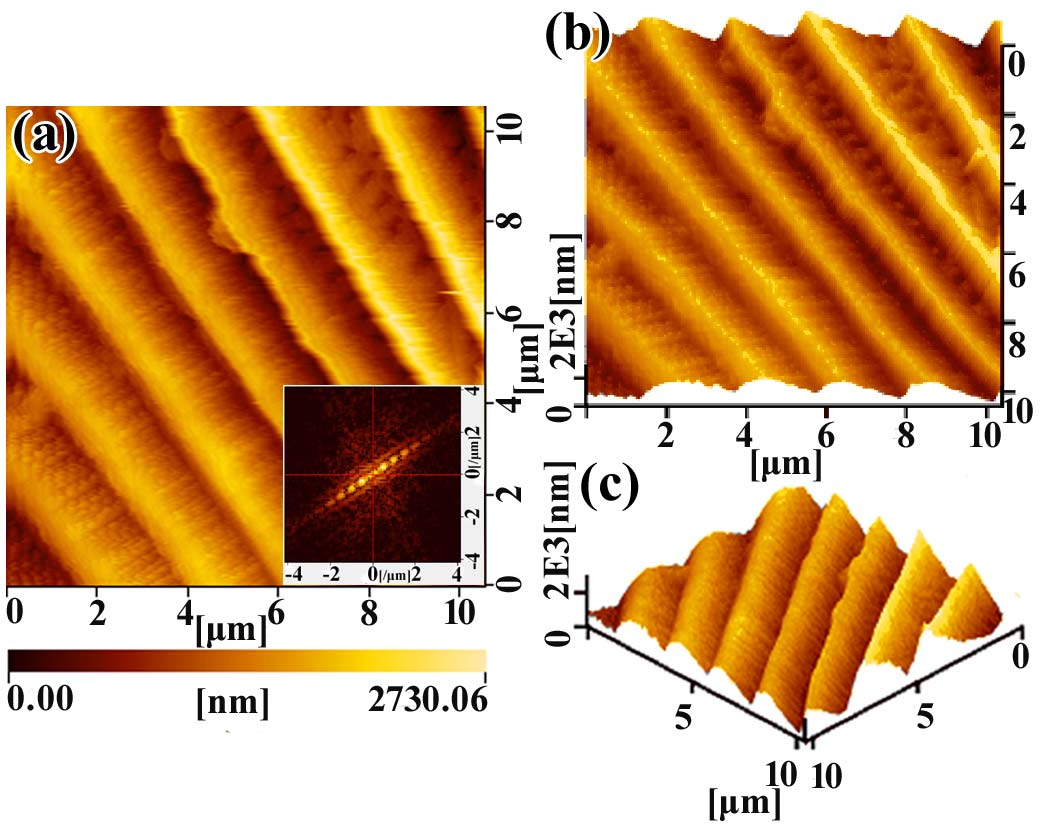
(a), (b) and (c) AFM images of CNMF_6h with different view angle. The inset of Figure 4(a) is the corresponding FFT images.

Typical TEM images of CNMF_6h are shown in Figure 3. The TEM micrographs provide further supporting evidence that the Ni NPs deposited onto the surface of the carbon-based SPTAS of the T_FW scale and assembled into a continuous film (Figure 3(a)). The lattice fringes, with an interplanar distance of d_Ni(111)_ = 0.203 nm, are evident in the HRTEM image of CNMF_6h (Figure 3(b)). Additionally, the SAED image displays both rings and dot patterns, corresponding to the major and minor phases in the product, respectively. The clear rings match well with the XRD results, and the relevant planes were indexed as (111), (200) and (220). The AFM images of CNMF_6h were recorded using a non-magnetic probe to perform a surface morphology analysis in tapping mode. The results of this analysis show that the Ni NPs deposited onto the periodic triangular roof-type ridges and that the periodic triangular roof-type antireflection structure was retained (Figure 4(a)). The fast Fourier transform (FFT) image further demonstrates that the structure of the CNMF_6h is a periodic structure (the inset of Figure 4(a)). As shown in Figure 4(b), the windows were also retained in CNMF_6h. In Figure 4(c), we can observe that the periodic triangular roof-type ridges form the periodic triangular roof-type antireflection structure and that the distance between adjacent ridges is approximately 2215 ± 39 nm, which is consistent with observations derived from SEM imaging (Figure 2(c)). By comparison with the AFM images of T_FW (Figure S4), Figure 4 reveals that the Ni NPs are deposited onto the carbon-based SPTAS surface of the T_FW scale and assembled into a film that successfully inherits the SPTAS. The differences between the SEM (Figure 2(c)) and AFM images (Figure 4) arise because the height of the ridges is greater than 2 μm, and deviations exist between the AFM image and the actual morphology of the CNMF_6h.

**Figure 5:**
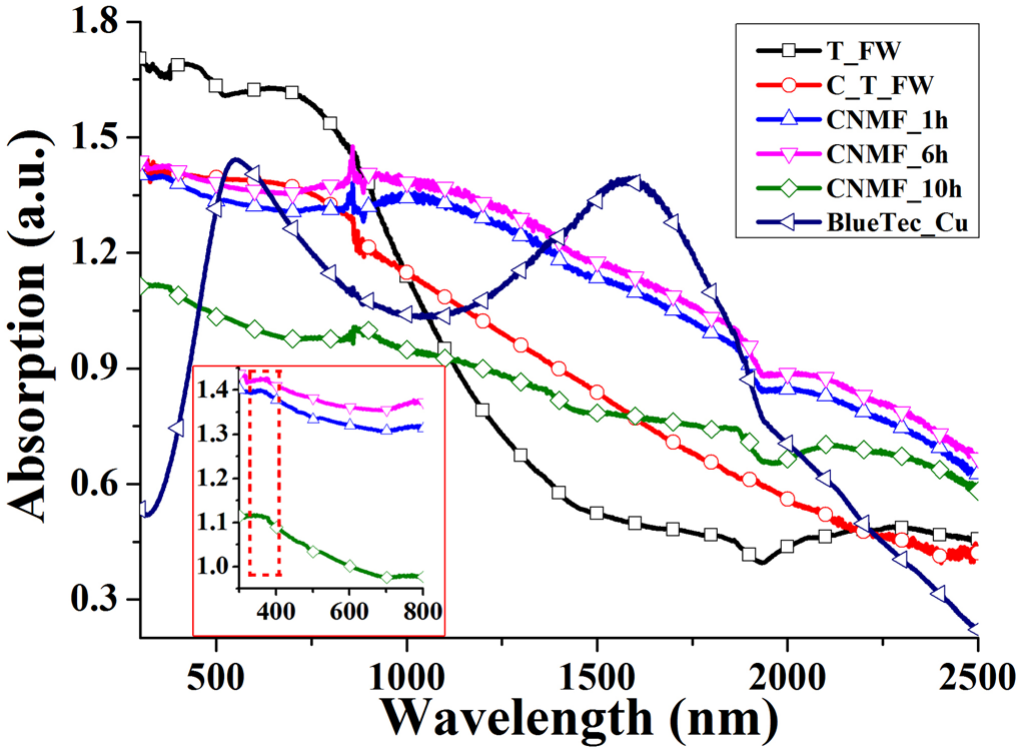
The absorption spectra over the wavelength range of 300–2500 nm of T_FW, carbonized T_FW (C_T_FW), the carbon-matrix Ni wing via Ni NP deposition for 1 h (CNMF_1h), 6 h (CNMF_6h), 10 h (CNMF_10h) and BlueTec_Cu. The inset is the absorption spectra of CNMF_1h, CNMF_6h and CNMF_10h over the wavelength range of 300–800 nm at a higher magnification.

The absorption spectra of the materials over the wavelength range of 300-2500 nm, are shown in Figure 5. The fluctuation of the absorption spectra at 860 nm is due to the transformation of the light source in the spectrophotometer. In the absorption spectra, we can observe that the T_FW exhibits superior light absorption capability in the visible light region because of the coupling effect between the melanin/chitin composite and the SPTAS^20^,^21^. Compared with the absorption properties of the T_FW, the carbonized T_FW (C_T_FW) shows an enhancement in absorption in the IR region, demonstrating that amorphous carbon provides better IR absorption than the melanin/chitin composite of the T_FW. Based on this conclusion, we decided to carbonize the chitin-matrix Ni film. The carbonization of the chitin-matrix Ni wings imbued them with enhanced broadband IR absorption due to the Ni NPs' plasmon resonance and the hybridization of adjacent Ni NPs' plasmon resonance^17^. As shown in the red dashed rectangular region in the inset in Figure 5, the absorption spectra exhibited characteristic peaks at 369 nm (CNMF_1h), 376 nm (CNMF_6h) and 377 nm (CNMF_10h), respectively, which could be attributed to the plasmon resonance of the Ni NPs^6^. This peak position redshifts with increasing time of the electroless deposition because a prolonged deposition time increases the diameter of the Ni NPs formed^6^,^24^. Among the CNMF_1h, CNMF_6h and CNMF_10h samples, CNMF_10h exhibited the weakest light absorption capability because the microstructures of the carbon-matrix Ni wing were almost filled by the excessive number of Ni NPs assembled on the wing surface (Figure S3). This finding indicates that the SPTAS of the T_FW scale is a key component of its enhanced light absorption properties. A shorter deposition time of 6 h induced a continuous layer of deposited Ni NPs to form on the main ridges, microribs and windows of the wings; consequently, the SPTAS was perfectly transferred to the film. However, an even shorter deposition time of 1 h produced a discontinuous layer of the Ni NPs on the surface of the SPTAS (Figure 2 and Figure S3). The CNMF_6h exhibited an effective broadband IR absorption because the Ni NP plasmon resonance and the hybridization of the adjacent Ni NPs' plasmon resonances were integrated with the carbon-matrix SPTAS. Furthermore, the absorption of the CNMF_6h was as much as 1.85 times greater than that of the T_FW over the wavelength range of 888–2500 nm. Compared with the absorption of BlueTec eta plus_Cu, the CNMF_6h exhibited a more effective light absorption performance over a wide spectral range, except in the wavelength ranges of 515–636 nm and 1393–1850 nm. The average absorbance intensities increased by 34.14% in the wavelength range of 300–2500 nm. BlueTec eta plus_Cu is a commercial absorber that functions as a solar thermal collector, which possesses an intense sunlight absorption performance and effectively transforms solar energy into heat (BlueTec GmbH & Co KG, Hesse Germany).

**Figure 6:**
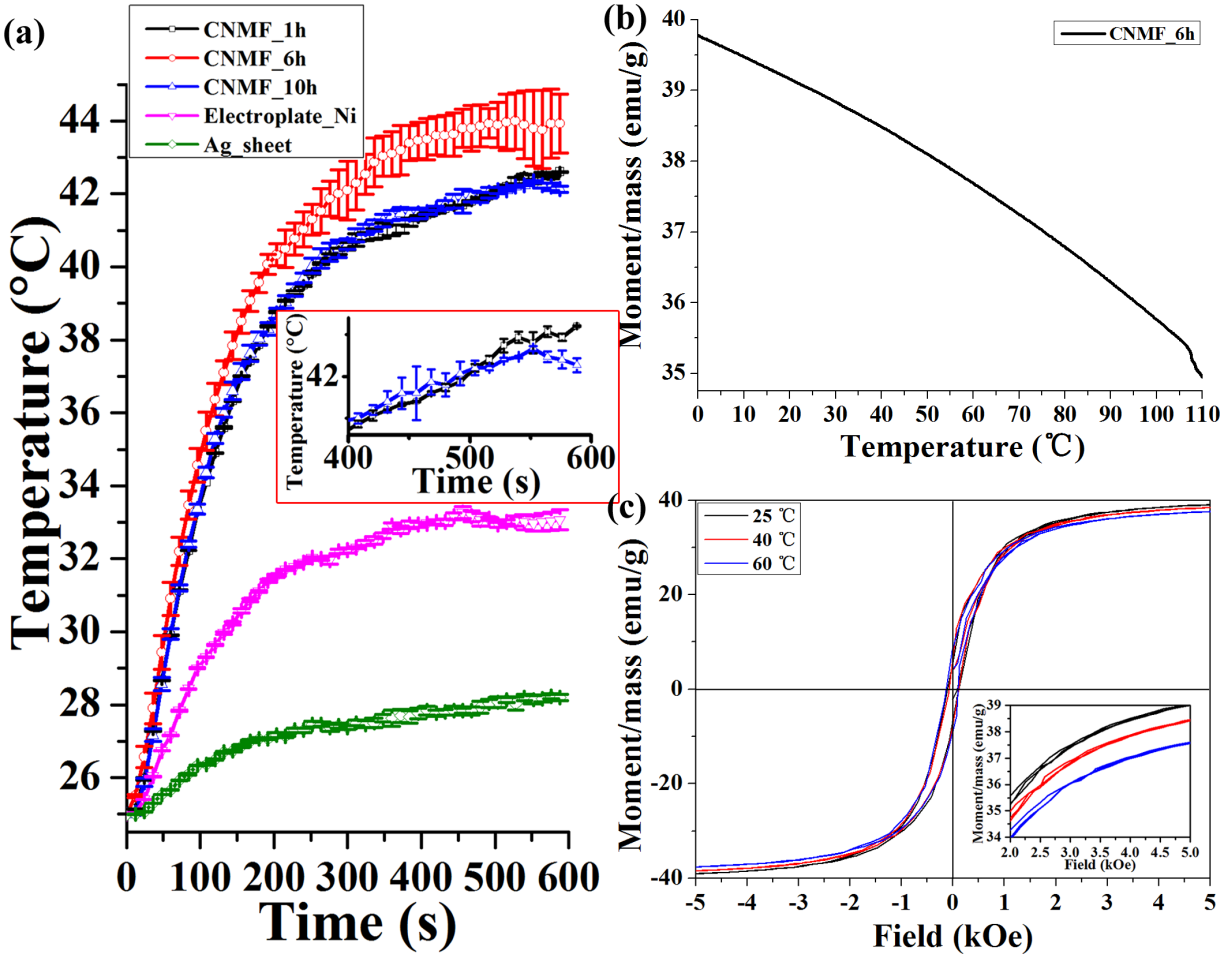
(a) The temperature elevation of CNMF_1h (via Ni NP deposition for 1 h), CNMF_6h (via Ni NP deposition for 6 h), CNMF_10h (via Ni NP deposition for 10 h), electroplating of the Ni NPs onto silver sheet (Electroplate_Ni) and the Ag sheet. The values were presented as the mean ± variance from triplicate samples. The inset of (a) is the temperature elevation of CNMF_1h and CNMF_10h over the time range of 400–600 s at a higher magnification. (b) Magnetization intensity versus temperature at the level of the magnetizing field H = 5000 Oe for the CNMF_6h. (c) Magnetic hysteresis loops of the CNMF_6h at the temperature of 25°C, 40°C and 60°C. The inset of Figure 6(c) is the magnetic hysteresis loops over the magnetic field range of 2–5 kOe at a higher magnification.

To study the photothermal conversion of the CNMF, the temperature elevation of the CNMFs (10 mm × 10 mm), which were affixed to a silver sheet with dimensions of 10 mm × 10 mm, were measured under irradiation by a 980 nm IR laser with a power density of 1.56 W/cm^2^. In comparison to the CNMF_1h, the CNMF_10h, the Electroplate_Ni (Figure S5) and the Ag sheet shown in Figure 6(a), CNMF_6h exhibited the greatest degree of IR photothermal conversion and the highest mean temperature elevation of up to 43.9°C. The reason for this enhanced performance of CNMF_6h is that it not only maintained the SPTAS of the T_FW but also deposited a sufficient number of Ni NPs onto the surface of the carbon-matrix SPTAS. Ni NPs have been demonstrated to have purely ferromagnetic nanostructures and to support plasmon resonance^6^,^26^. Consequently, the CNMF_6h provided clear infrared photothermal conversion enhancement that could be ascribed to the effectiveness of coupling between the Ni NPs' plasmon resonances and the carbon-based SPTAS. The Electroplate_Ni was obtained by electroplating Ni NPs onto a silver sheet (see Experimental Section in the Supporting Information for further details regarding the fabrication route). The temperature elevation curves of CNMF_1h and CNMF_10h almost overlapped, although the CNMF_10h exhibited a lower light absorption capability. This overlapping occurred because the CNMF_10h sample contained more photothermal conversion material. Thus, the degree of photothermal conversion not only determined by the light absorption property, but also dictated by the amount of photothermal conversion material present. As shown in the inset of Figure 6(a), the temperature elevation fostered by CNMF_1h is ultimately higher than that of CNMF_10h. Consequently, to obtain the maximum photothermal conversion performance, a material must not only have a superior light absorbing structure, but also possess sufficient photothermal conversion material. Indeed, the CNMF_6h combined sufficient photothermal conversion material (Ni NP coating) with the carbon-matrix SPTAS inherited from T_FW which possesses superior light absorption characteristics. The effective IR photothermal conversion of the CNMF_6h was also demonstrated by comparing it with the solar photothermal conversion of nanocomposite films consisting of Ni nanoparticles embedded into diamond-like carbon (DLC:Ni)^27^. The temperature increase of DLC:Ni is only approximately 20°C when illuminated by a 20 kW/m^2^ concentrated solar simulator light. The mean temperature increase of CNMF_6h is much as 18.9°C when illuminated by a 980 nm infrared laser with a power density of 1.56 W/cm^2^. In Figure 6(b), the magnetization intensity gradually decreases as the temperature of the CNMF_6h increases, an effect that can be induced by the incident IR irradiation. From Figure 6(a) and (b), we may observe that the 980 nm IR irradiated on the surface of CNMF_6h induced a photothermal effect, resulting in temperature elevation and ultimately leading to magnetic variation. These effects result from an increase in the distance between atoms with increasing temperature, which reduces the magnitude of the exchange interaction between atoms. Meanwhile, the thermal motion destroys the regular orientation of the magnetic moment of the atoms, decreasing the spontaneous magnetization intensity. The magnetic hysteresis loops of the CNMF_6h at temperatures of 25°C, 40°C and 60°C are presented in Figure 6(c). The magnetic hysteresis loops show a clearly decreased saturation magnetization with an increase in the temperature (Figure 6(c)). The decrease factor is in agreement with the decrease of magnetization in the image of magnetization intensity versus temperature (Figure 6(b)). However, almost no change occurred in the coercive forces. In short, the 980 nm IR excitation induces a temperature increase that results in a reduction of the saturation magnetization of the CNMF_6h. Consequently, the CNMF_6h achieves optimizational magnetoplasmonic integration.

**Figure 7:**
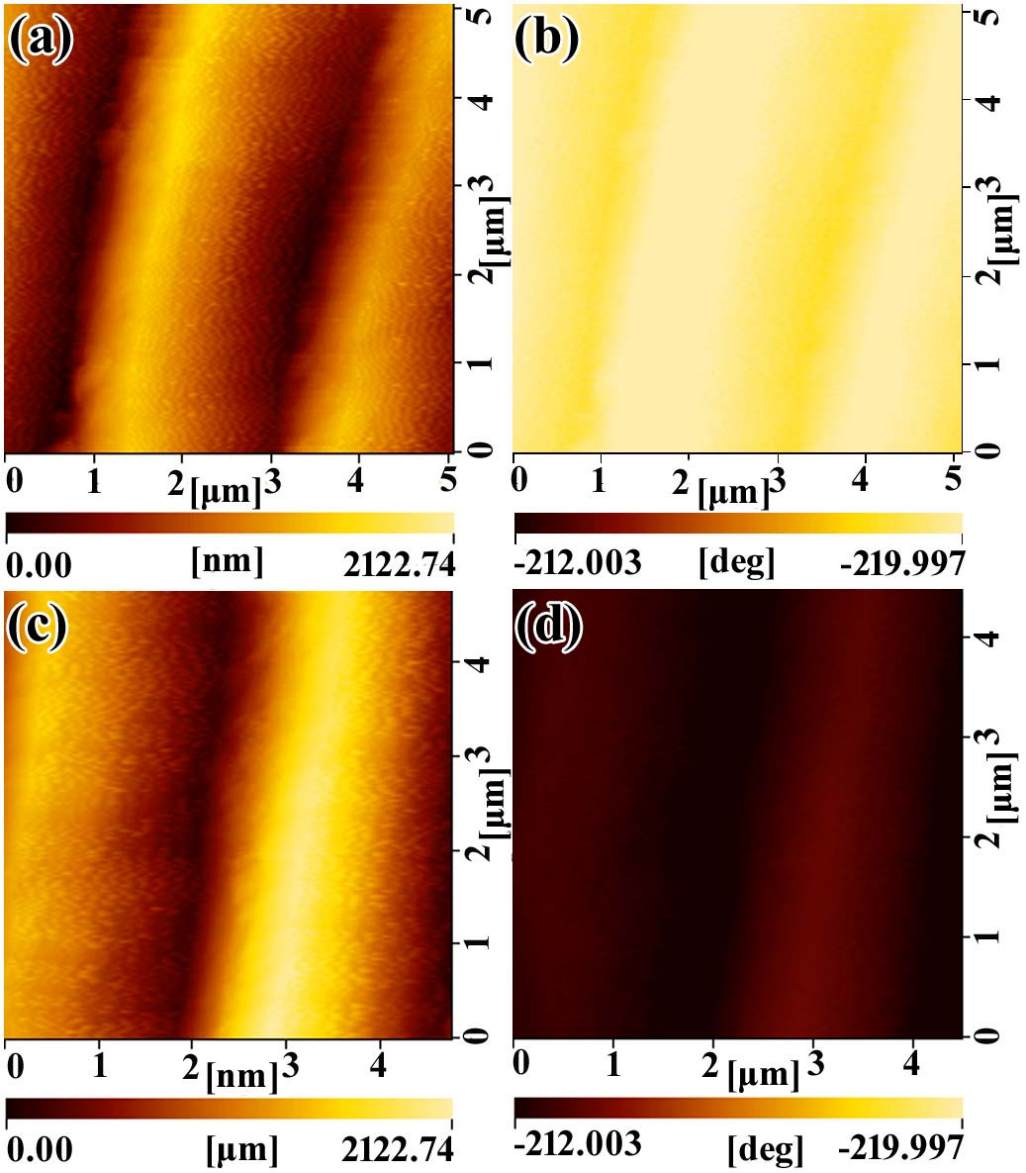
AFM-MFM images performed using a magnetic probe on CNMF_6h. (a) and (c) are the AFM images of CNMF_6h at the temperatures of 25°C and 40°C, respectively. (b) and (d) are the MFM images of CNMF_6h at the temperatures of 25°C and 40°C, respectively.

As shown in Figure 7, AFM and MFM were used to characterize the structural and magnetic properties of the CNMF_6h, respectively. A magnetic probe was used to detect the surface morphology in AFM and MFM modes, and the probe was lifted to enable scanning at a fixed height (500 nm) above the sample. Figure 7(a) and (c) show typical AFM scans with the ridges of T_FW. However, compared with Figure 4, the resolution of Figure 7 is lower because the magnetic probe was lifted to enable scanning at a fixed height (500 nm) above the sample. In these MFM images (Figure 7(b) and (d)), the magnetic structure of the scanned areas is also evident, and the brighter regions correspond to the higher magnetic areas. The clearly magnetized areas, or areas of magnetic percolation, are located on the ridges of T_FW. Figure 7(b) and (d) show typical MFM scans with ridges of T_FW. The topography of the MFM images is consistent with the morphology recorded using AFM imaging. The reason is that the magnetic dipole is centered on the magnetic Ni NP, and the ridge pattern arises that can be attributed to the intrinsic magnetic properties of the Ni NPs that cover the surface of the SPTAS of T_FW scales^28^,^29^,^30^. Good correlations were observed between the AFM images (Figure 7(a) and (c)) and the MFM images (Figure 7(a) and (c)). The bright magnetic areas occur when magnetic sample interacted with the tip^31^,^32^. In contrast, the brightness of the window regions of the SPTAS were darker than that of the ridge regions, which indicates that the window regions did a weaker interact with the tip because the distance between the magnetic tip and the magnetic surface of the windows of the SPTAS is too large to effectively generate the interaction. Consequently, the results in Figure 7 clearly suggest that the surface of the SPTAS of CNMF_6h exhibits strong magnetic properties^33^, which results from the magnetic dipoles of the Ni NPs, and the magnetic dipole interactions of the Ni NPs and between adjacent ridges^34^. Compared with Figure 7(b), the color of Figure 7(d) is darker, which demonstrates that the magnetic properties weaken when the temperature increased from 25°C to 40°C.

## Conclusions

In summary, we explored a straightforward and low-cost method for fabricating carbon-matrix nickel composite magnetoplasmonic films with SPTAS on a macroscopic centimeter-scale, in which purely ferromagnetic nanostructures support plasmon resonance. The thickness of the Ni NPs deposited onto the T_FW and the morphology of the carbon-matrix Ni wing were controlled via varying the electroless deposition time. CNMF_6h exhibited enhanced IR absorption over a broad wavelength range because of the Ni NP plasmon resonance and the hybridization of the adjacent Ni NP plasmon resonances integrated with the carbon-based SPTAS. The enhanced absorption of the CNMF_6h was as much as 1.85 times greater than that observed for T_FW over the wavelength range of 888–2500 nm. When a 980 nm IR laser was used to irradiate the surface of the CNMF_6h, the IR photothermal effect resulted in an increase in temperature. Meanwhile, a decrease in the magnetization intensity and a reduction of the saturation magnetization arose, which resulted from the temperature increase during the IR irradiation. The good correlation between the AFM and MFM images demonstrate that the surface of the SPTAS of CNMF_6h exhibited strong magnetic properties. The reported route is promising for the design of novel magnetoplasmonic films with potential applications in IR information recording and in heat-assisted magnetic recording via IR excitation.

## Methods

### Materials

*Troides helena* butterflies were obtained from Shanghai Natural Wild-Insect Kingdom Co., Ltd. Absolute ethanol (EA, 97%) was purchased from Changshu Yangyuan Chemical Co., Ltd (Changshu, Jiangsu, China). Nitric acid (NA, 67%), ethylenediamine (ED, 99%), nickel(II) sulfate hexahydrate (NiSO_4_·6H_2_O), lactic acid, ammonium hydroxide (NH_3_·H_2_O), and silver sheet (0.1*50 mm) were purchased from Sinopharm Chemical Reagent Co., Ltd (Shanghai, China). Borane dimethylamine complex (BD, 96%) was purchased from Aladdin Industrial Co., Ltd (Shanghai, Chinas). All of these chemicals were analytically pure and were used as received without further purification. Solamet PV416 was purchased from Du Pont China Holding Co., Ltd (Shanghai, China). The BlueTec eta plus_Cu and the Cu substrate were purchased from BlueTec GmbH & Co KG (Hesse, Germany).

### Fabrication of CNMF

To aminate the T_FW, T_FW was firstly immersed in EA for 15 min to wash the dust off the surface, and rinsed in deionized water. The T_FWs were then immersed in dilute 8 vol% NA for 2 h and rinsed in deionized water. Next, the T_FW was immersed in an ethanol solution of ED (25 vol%) for 6 h to aminate the T_FW, washed with EA, then rinsed in deionized water^23^. Ni deposition was achieved by immersing the aminated T_FW into a Ni electroless plating solution at 50°C, rinsing it with deionized water, and drying in vacuum at 30°C. The deposition solution consisted of nickel sulfate (4 g), sodium citrate (2 g), lactic acid (1 g), dimethylamine borane (0.2 g), deionized water (100 ml) and ammonium hydroxide (3 ml), with pH 6.8^23^. The thickness of Ni NPs deposited onto the T_FW is controllable by controlling the deposition time, as shown in Figure S1. In the end, the chitin-matrix Ni wing of T_FW was carbonized using a vacuum tube furnace at 450°C for 1 h with a heating rate of 3°C·min^−1^ from room temperature under the vacuum condition. Consequently, the CNMF was fabricated.

### Sample Characterization

Characterization using scanning electron microscopy (SEM) was performed on a 20-kV field emission SEM instrument (Quanta 250, FEI, Hillsboro, OR, USA) equipped with an energy dispersive spectrometer (EDS, 80 mm^2^ detector, Oxford Instruments, Abingdon, UK). X-ray diffraction (XRD) measurements were conducted using a Rigaku D/max-2550 instrument equipped with a Cu-Kα radiation source (Rigaku Corp., Tokyo, Japan). Transmission electron microscopy (TEM), high resolution transmission electron microscopy (HRTEM) and selected area electron diffraction (SAED) measurements were performed on a JEM-2100F transmission electron microscope (JEOL, Peabody, MA, USA) operated at an acceleration voltage of 200 kV. For these analyses, the samples were dispersed in ethanol using an ultrasonic wave cleaner and were then drop-cast onto carbon-supported Cu grids. The absorption over the wavelength range of 300–2500 nm was measured using a Lambda 750 UV-VIS-NIR spectrophotometer (PerkinElmer, Waltham, MA, USA). For measurement of the photothermal conversion, a 980 nm NIR laser with a power density of 1.56 W/cm^2^ was vertically emitted downward toward the surface of the CNMF (10 mm × 10 mm), which was affixed onto a silver sheet using Solamet PV416. The size of the silver sheet was 10 mm × 10 mm. The light source was a semiconductor laser with an externally adjustable power source (0–2 W) and an adjustable laser module diameter (4–10 mm) (Shenzhen Yatai Optoelectronic Technology Co., Ltd., Shenzhen China). 980 nm IR lasers have been widely used to measure photothermal conversion performance^35^,^36^,^37^,^38^. In the work reported here, the diameter of the laser beam was fixed at 5 mm. The output power was independently calibrated using a VLP-2000MW laser power meter. A thermal resistor (Pt-100) with a readout accuracy of ± 0.1°C was attached to the backside of the silver sheet. Magnetization measurements at different temperatures were performed using a PPMS-9 physical property measurement system manufactured by Quantum Design (USA). Atomic force microscopy (AFM) images and magnetic force microscopy (MFM) images were recorded using an environment control scanning probe microscope (SII Nanonavi E-Sweep, USA) under ambient conditions.

## Author Contributions

J.L.T., W.Z. and D.Z. conceived and designed the experiments. J.L.T. performed all the experiments and analyzed all the data. Y.Q.H., Y.H.W. and Z.J.Z. helped analyze the TEM and XRD data. J.L.T. and W.Z. co-wrote the paper. Q.L.L. and D.Z. discussed the results and commented on the manuscript.

## Supplementary Material

Supplementary Information Supplementary Information of manuscript
