# Supplementary material for: Infrared-induced variation of the magnetic properties of a magnetoplasmonic film with a 3D sub-micron periodic triangular roof-type antireflection structure
Source: Sci Rep. 2015 Jan 26;5:8025. doi: 10.1038/srep08025 (PMC4306118; doi:10.1038/srep08025)
Supplement: Supplementary Information — of manuscript [file srep08025-s1.pdf]

Supplementary Information

Infrared-induced variation of the magnetic properties of a magnetoplasmonic film with a 3D sub-micron periodic triangular roof-type antireflection structure

Junlong Tian[1], Wang Zhang[1], Yiqiao Huang[1], Qinglei Liu[1], Yuhua Wang[2], Zhijian Zhang[3] & Di Zhang[1]

[1]State Key Laboratory of Metal Matrix Composites, Shanghai Jiao Tong University, 800 Dongchuan Road, Shanghai, 200240, P. R. China, [2]Department of Prosthodontics, Shanghai Jiao Tong University, 800 Dongchuan Road, Shanghai, 200240, P. R. China, [3]Jushi Fiberglass Research Institute, Zhejiang Key Laboratory for Fiberglass, Research Jushi Group Co., Ltd., Zhejiang, 314500, P. R. China.
Correspondence and requests for materials should be addressed to W.Z (wangzhang@sjtu.edu.cn) or D.Z (zhangdi@sjtu.edu.cn)

Experimental details

Detailed fabrication route

The Electroplate_Ni was obtained by electroplating of the Ni NPs onto silver sheet. In order to remove the chemical absorbate on the Ag plate surface, each sample sheet was cleaned and anodized at 0.25 A ·cm^{-2} in a solution (10 g ·L^{-1}KOH, 10 g ·L^{-1} santomerse, 70 g ·L^{-1} deoil powder) for 20 s, rinsed with pure water. Ni coating was electroplated at a constant current density of 3 A/cm^2 for 5 min. The electrolyte was composed of analytical pure $NiSO_4 \cdot 6H_2O$(250 g/L), $NiCl_2 \cdot 6H_2O$ (40 g/L), H_3BO_4 (40 g/L), Saccharin (1 g/L), 2-Butyne-1,4-diol (0.5 g/L), Sodium dodecyl sulfate (0.1 g/L). The bath temperature was maintained at 50-60 ℃.

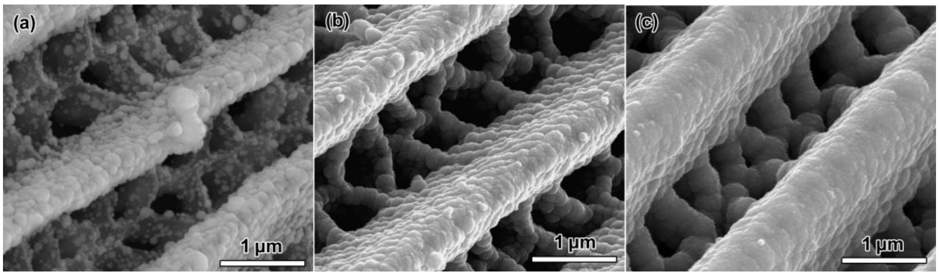

Figure S1. SEM images of chitin-matrix Ni wing of T_FW fabricated via Ni NPs deposition for (a) 1 h (chitin-matrix Ni wing_1h), (b) 6 h (chitin-matrix Ni wing_6h) and (c) 10 h (chitin-matrix Ni wing_10h).

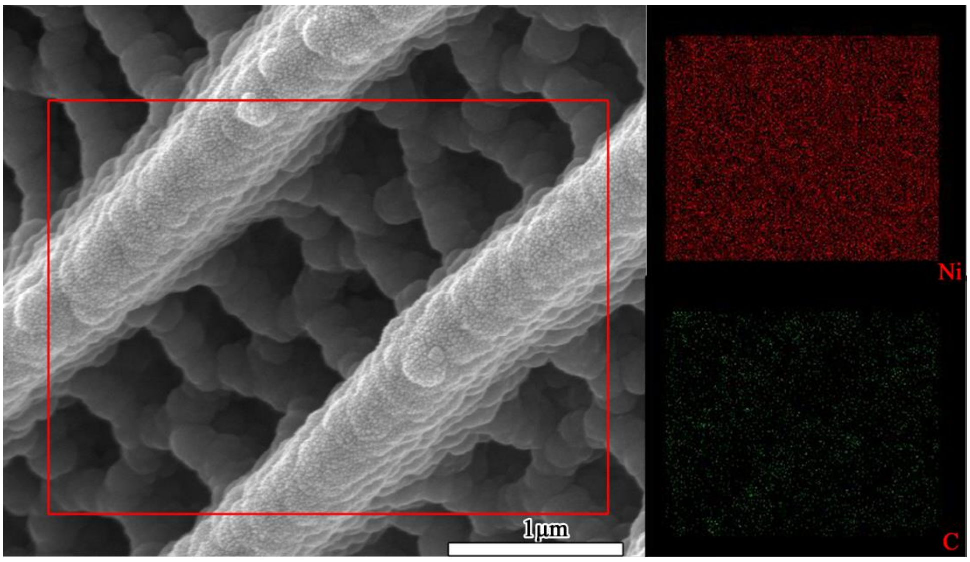

Figure S2. SEM image of CNMF_6h and elemental map showing the distribution of Ni and C atoms on the surface of the SAQCS of the T_FW scales.

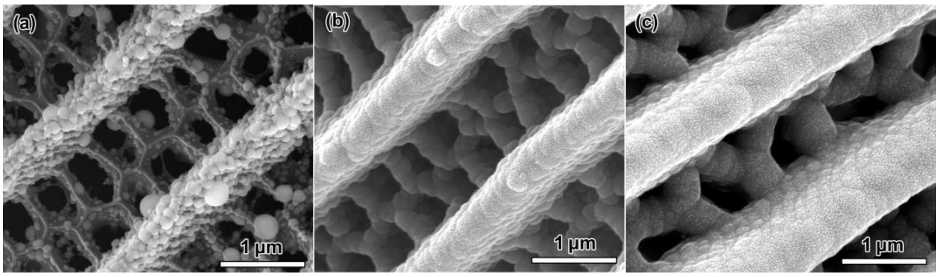

Figure S3. SEM images of carbon-matrix Ni wing via Ni NPs deposition for (a) 1 h (CNMF_1h), (b) 6 h (CNMF_6h) and (c) 10 h (CNMF_10h).

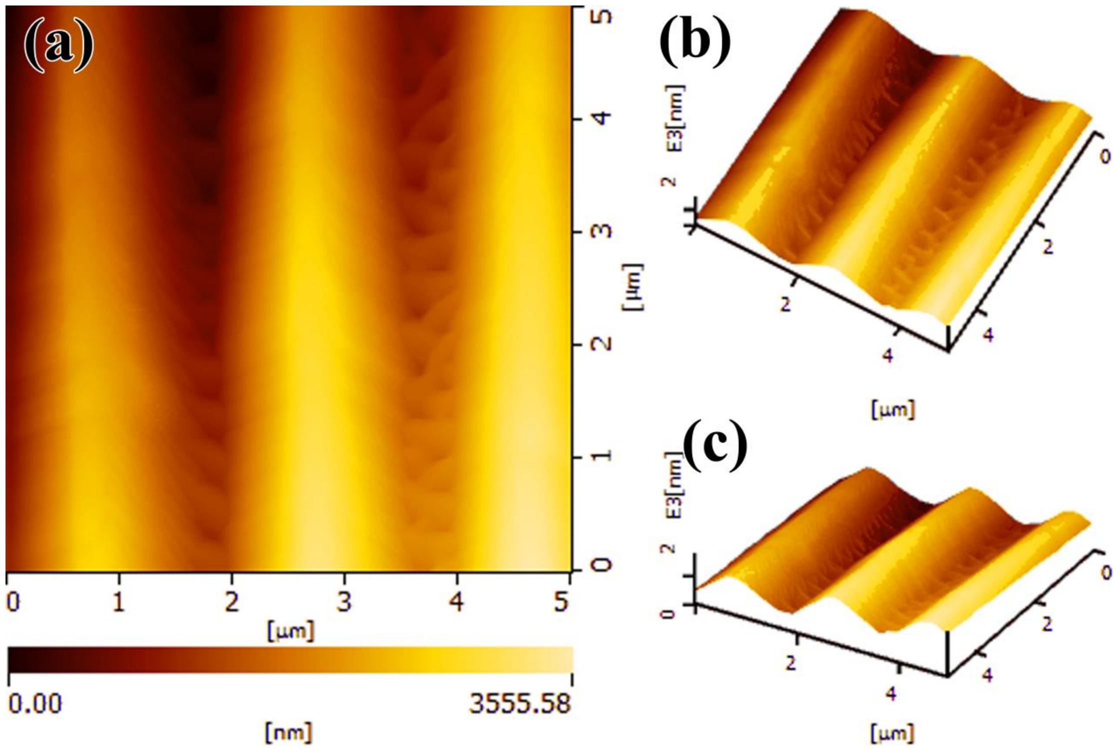

Figure S4. (a), (b) and (c) AFM images of T_FW with different view angle, respectively.

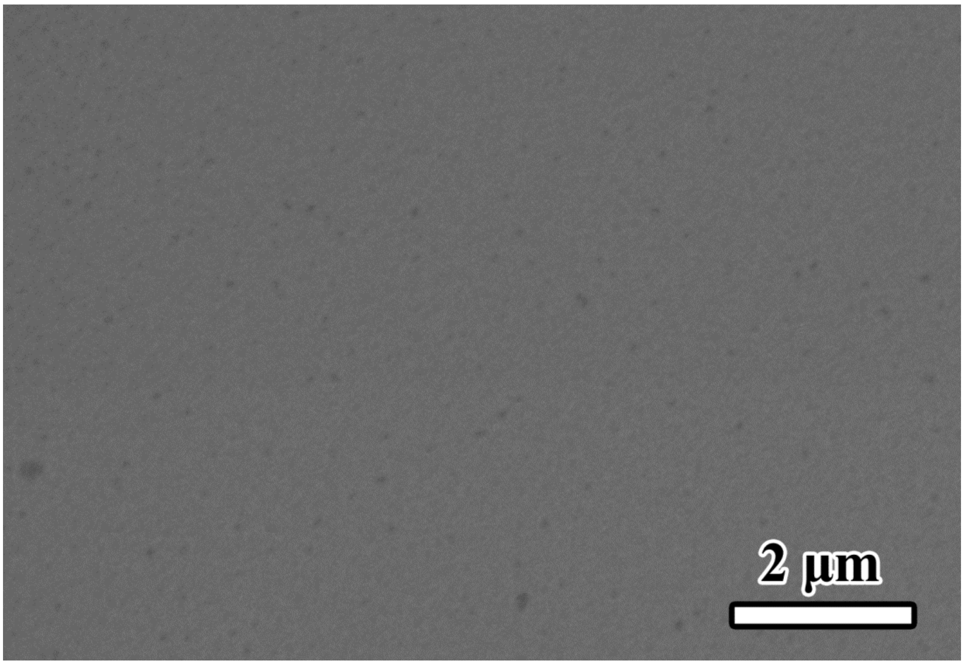

Figure S5. SEM image of the Electroplate_Ni (electroplating of the Ni NPs onto silver sheet).
